# Antibiotic Stewardship Implementation at Hospitals Without On-Site Infectious Disease Specialists: A Mixed-Methods Study

**DOI:** 10.1017/ash.2021.127

**Published:** 2021-07-29

**Authors:** Daniel Livorsi, Eli Perencevich, Kenda Stewart Steffensmeier, Matthew Goetz, Heather Reisinger

## Abstract

**Background:** Hospitals are required to have antibiotic stewardship programs (ASPs), but there are few models for implementing ASPs without the support of an infectious disease (ID) specialist, defined as an ID physician and/or ID pharmacist. In this study, we sought to understand ASP implementation at hospitals within the Veterans’ Health Administration (VHA) that lack on-site ID support. **Methods:** Using a mandatory 2016 VHA survey, we identified acute-care hospitals that lacked an on-site ID specialist. For each hospital, antibiotic use (2018–2019) was quantified as days of therapy (DOT) per 1,000 days present, based on NHSN methodology for tracking all antibacterial agents. From July 2019 through April 2020, we conducted semistructured interviews with personnel involved in or affected by ASP activities at 7 qualifying hospitals. All interview transcripts were analyzed using thematic content analysis. **Results:** Of the 7 acute-care hospitals, 6 (86%) had a long-term care unit; 3 (43%) had an intensive care unit; and 2 (29%) had full-time employment equivalents dedicated to stewardship. Sites averaged 1,075 (SD, ±654) and 148 (SD, ±96) admissions per year in acute-care and long-term care, respectively. At the site-level, mean antibiotic use was 486 DOT (SD, ±98) per 1,000 days-present in acute-care and 207 DOT (SD, ±74) per 1,000 days present in long-term care. We interviewed 42 personnel across the 7 sites. Although sites reported using similar interventions to promote antibiotic stewardship, the shape of these interventions varied. The following 4 common themes were identified: (1) The primary responsibility for ASPs fell on the pharmacist champions, who were typically assigned multiple other non-ASP responsibilities. (2) The pharmacist champions were more successful at gaining buy-in for stewardship initiatives when they had established rapport with clinicians, but at some sites, the use of contract physicians and frequent staff turnover were potential barriers. (3) Some sites felt that having access to an off-site ID specialist was important for overcoming institutional barriers to stewardship and improving the acceptance of their stewardship interventions. (4) In general, stewardship champions struggled to mobilize institutional resources, which made it difficult to advance their programmatic goals. **Conclusions:** In this study of 7 hospitals without local ID support, we found that ASPs are largely a pharmacy-driven process. Remote ID support, if available, was seen as helpful for implementing stewardship interventions. These findings may inform the future implementation of ASPs in settings lacking local ID expertise.

**Funding:** No

**Disclosures:** None

